# A psycho-educational model for university lecturers to facilitate the constructive management of aggression

**DOI:** 10.4102/hsag.v25i0.1363

**Published:** 2020-11-06

**Authors:** Rika R. Toerien, Chris P.H. Myburgh, Marie Poggenpoel

**Affiliations:** 1 Department of Educational Psychology, Faculty of Education, University of Johannesburg, Johannesburg, South Africa; 2 Department of Nursing, Faculty of Health Sciences, University of Johannesburg, Johannesburg, South Africa

**Keywords:** experienced aggression, implementation, lecturers, psycho-educational model, university, workshop

## Abstract

**Background:**

Experienced workplace aggression is a lingering phenomenon and comprises an extensive body of knowledge. Experiences of workplace aggression require constructive intervention and support. These interventions and support must aim to assist university lecturers to constructively manage experiences of aggression.

**Aim:**

The aim of this article is to describe the process followed to develop, describe and evaluate a psycho-educational model for university lecturers that could be used as a conceptual framework of reference to facilitate the constructive management of experienced aggression.

**Setting:**

The model is applicable in universities where the university lecturers work and experience aggression. This study was conducted in a specific college at a university in Johannesburg in South Africa.

**Method:**

A theory-generating, qualitative, exploratory, descriptive and contextual design was applied to develop a psycho-educational model. The process included four steps: concept analysis, relationship statements, description of the model and evaluation of the developed model. The criteria of clarity, simplicity, generality, accessibility and importance were used during the evaluation of the model.

**Results:**

The central concept in this study was to facilitate constructive management. The psycho-educational model as the conceptual framework of reference for facilitating the constructive management of experienced aggression was described and evaluated. University lecturers’ destructive management of experienced aggression formed the basis for the psycho-educational model.

**Conclusion:**

The psycho-educational model provides a conceptual framework of reference for university lecturers that may assist them to constructively manage experiences of aggression in their places of work.

## Introduction

Experiences of aggression are part of individuals, society and the workplace realities of today. Aggression is not a singular or unitary term but has different meanings and occurs in many different forms (Breet, Myburgh & Poggenpoel 2010:511; Hills, Lama & Hills 2018:607–612; Kelloway et al. 2010:18–19). Individuals who are targeted in the workplace often develop physical and emotional health problems and may feel despondent (Murrell 2018:n.p.; Rogojan 2009:50). Unfortunately, universities have not escaped this social tendency and phenomenon. Toerien (2014:65–83) discovered that aggression – hidden in frustration – is experienced by university lecturers on different levels and in many forms within their work environments. In addition to the various levels and forms of aggression, the university lecturers also indicated that they often implement negative coping strategies to manage the experienced aggression. Finally, university lecturers’ lived experiences of aggression occur on personal, interpersonal and systemic levels and influence the lecturers’ professional and personal development, interpersonal relationships, work performance and well-being.

The aforementioned results provided the foundation for the development of the psycho-educational model for university lecturers to facilitate the constructive management of experienced aggression in this study. Employees who receive support from their organisation often feel more in control and express positive feelings (Everton, Jolton & Mastrangelo 2009:53). As a result, employees who feel more in control of experienced aggression will experience better job satisfaction and professional effectiveness, as well as physical and mental health (Loh, Restubog & Zagenczyk 2010:236). University lecturers’ daily encounters with diverse challenges and experiences of aggression often culminated in destructive management of experiences of aggression and therefore necessitated the need for support, knowledge and skills development, as well as clear institutional guidelines. These needs and destructive management of experienced aggression translated into the question ‘what can be done to support university lecturers to constructively manage experienced aggression?’, which guided the development of a psycho-educational model. The argument is that the constructive management of experienced aggression by university lecturers is facilitative of a personal and professional growth process that may contribute to the development of intrapersonal, interpersonal and aggression management skills and potential. Therefore, the development of a psycho-educational model is required for university lecturers to facilitate the constructive management of their experienced aggression (Toerien 2019:7–9). This article contributes to research that focuses on and aims to develop psycho-educational models for experienced aggression in the workplace.

The purpose of this study was to describe the process followed to develop, describe and evaluate a psycho-educational model for university lecturers that could be used as a conceptual framework of reference to facilitate the constructive management of experienced aggression.

## Research design and method

### Design

A theory-generating design that is qualitative, exploratory, descriptive and contextual was used to develop the psycho-educational model (Chinn & Kramer 2015:156–158; Creswell 2014:8; Denzin & Lincoln 2011:3; Walker & Avant 2011:3–4). The qualitative research design allowed for a multifaceted, in-depth and holistic inquiry of the phenomenon to facilitate constuctive management, (Nieuwenhuis 2014:51). The exploratory design also allowed for an investigative approach to the phenomenon to gain deep and unique insight into the phenomenon (Grove, Gray & Burns 2015b:21). The descriptive design aimed to give an accurate and complete description of the phenomenon and its characteristics and qualities (Chinn & Kramer 2015:247; Polit & Beck 2014:552). The contextual design further aimed at an understanding of the phenomenon in the natural context of the South African university boundaries and data valid in this specific context (Botma et al. 2010:128; Creswell 2014:185; eds. De Vos et al. 2011:64).

The study’s theory-generating design provided an inductive and dynamic process through which the phenomenon was systematically and logically studied, clearly defined concepts developed and explained, and relationships described to move to model development and theory (Chinn & Kramer 2015:220; Lunenberg 2011:2; Walker & Avant 2011:3). The goal of this study was to develop, describe and evaluate a psycho-educational model for university lecturers to facilitate the constructive management of their experienced aggression.

### Method

The psycho-educational model development was conducted using the four steps of Chinn and Kramer (2015:154–227). The four steps are concept analysis, relationship statements, description of the model and evaluation of the developed model (Chinn & Kramer 2015:154–244).

#### Step 1: Concept analysis

Concept analysis was completed using Walker and Avant’s (2011:157–174) eight-step method. The specific details will be discussed in a possible future article. The eight steps are presented in three subdivisions of concept analysis, which are concept identification, definition of concepts and concept classification. Description of the three subdivisions follows.

**Concept identification:** The central concept resulted from the researcher’s master’s dissertation findings (Toerien 2014), which explored university lecturers’ experiences of aggression in a faculty at a university. In the study, qualitative data collection was conducted through one-on-one phenomenological interviews, followed by data analyses using Tesch’s method of descriptive analysis (Creswell 2009:184; Tesch 1990).

**Concept definition:** The essential and related attributes of the central concept, facilitate constructive management, were defined using various dictionary definitions and definitions from subject literature (Walker & Avant 2011:162). These essential and related attributes were applied in the development and description of the model in this study (Walker & Avant 2011:163).

**Concept classification:** The survey list of Dickoff, James and Wiedenbach (1968:415) was applied to classify the following concepts inclusive of the agent (facilitator), recipient (university lecturers), context (framework), dynamics, process and the terminus.

#### Step 2: Relationship statements

The concepts identified and defined in step 1 were placed into related relationships with each other, and descriptive relationship statements were presented (Chinn & Kramer 2015:182). These interrelated statements were fundamental to the form and function of the psycho-educational model.

#### Step 3: Description of the model

A psycho-educational model was developed and described by implementing Chinn and Kramer’s (2015:187–198) six guidelines for the description and critical reflection of empirical theory. These guidelines include purpose, conceptual meaning, relationships, theoretical structure and assumptions. A visual model was designed to describe the structure and process of the psycho-educational model.

#### Step 4: Evaluation of the model

The model evaluation was guided by the criteria of clarity, simplicity, generality, accessibility and importance of the model (Chinn & Kramer 2015:199–206). The psycho-educational model was evaluated by a panel of academics who were experienced in model development and evaluation as well as peers with experience in theory-generating research and model development. The model was found to be compliant with the preceding criteria for model development.

### Measures to ensure trustworthiness

Guba’s (1981) four components of trustworthiness (truth value, applicability, consistency and neutrality) were observed in this study. Lincoln and Guba’s (1985:301–331) model for trustworthiness criteria, in line with the study’s philosophy, principles and qualitative inquiry, were used to measure these four components. The criteria used were credibility, transferability, dependability and confirmability. These measures to ensure trustworthiness were implemented during the data analysis and concept analysis of the central concept, to facilitate constructive management, in step 1. The evaluation panel also applied these measures in step 4, ‘Evaluation of the model’.

### Ethical considerations

The researchers observed four ethical principles throughout the research, data collection and data management for the concept analysis as stipulated by the general ethical principles of the South African Medical Research Council (2006), namely, autonomy, non-maleficence, beneficence and justice (Adams & Callahan 2013:n.p.; Dhai & McQuoid-Mason 2011:43–44). Ethical clearance was received from the Academic Ethics Committee of the Faculty of Education at the university where the study was conducted (ethical clearance number: 2016–086). Permission was also granted by the specific faculty’s management and faculty leader team of the university where the study was conducted. All ethical principals were strictly adhered to, and the autonomy of individuals during the primary data collection was respected.

## Results

The results of the developed psycho-educational model are described according to Chinn and Kramer’s guidelines (2015:186–198). These guidelines include the purpose, underlying assumptions, context, theoretical definitions of concepts, relationship statements and structure of the model.

### Purpose of the psycho-educational model

The purpose of this psycho-educational model was to serve as a conceptual framework of reference for university lecturers to facilitate the constructive management of experienced aggression. The purpose statement of the psycho-educational model in context implies actions to be taken by both the university lecturers, as well as the psycho-educational facilitator, to promote development and increase the university lecturers’ effectiveness in managing experienced aggression constructively as a long-term goal (Chinn & Kramer 2015:188). The following assumptions shaped the structure of the model.

### Assumptions of the psycho-educational model

The assumptions of the psycho-educational model are grounded in the theory of health promotion (University of Johannesburg 2017:2–16). This model has several primary assumptions:

- The facilitation of constructive management of experienced aggression is a process that the psycho-educational facilitator makes easier for university lecturers.
- The psycho-educational facilitator assists university lecturers to increase their effectiveness at managing their experiences of aggression.
- This increased effectiveness is helpful to the university lecturers and promotes their development.
- With their promoted development and increased effectiveness, university lecturers should be able to deal with and control their experiences of aggression.

Further assumptions derive from the primary assumptions:

- During the facilitation process, university lecturers can discover and gain knowledge and skills through self-directed learning to increase their effectiveness at managing experiences of aggression constructively.
- The facilitator aims to assist university lecturers in identifying and understanding experiences of aggression in the university context and in discovering and gaining knowledge and skills that are helpful to them in dealing with experienced aggression more constructively. This specific aim promotes the development of university lecturers and increases their effectiveness at managing experienced aggression.
- University lecturers should willingly, actively and interactively participate in the facilitative interaction.
- The facilitation of constructive management of experienced aggression will contribute to university lecturers’ personal and professional development.
- The facilitation of constructive management of experienced aggression will contribute to university lecturers’ improved intrapersonal and interpersonal relationships.
- The facilitation of constructive management of experienced aggression will contribute to university lecturers’ improved communication and aggression management strategies.
- The facilitation of constructive management of experienced aggression may indirectly contribute to improved mental health for university lecturers.

### Context where the psycho-educational model applies

The model is applicable in the universities where the university lecturers work and experience aggression. In this study, it is a college at a university in Johannesburg, South Africa.

### Theoretical definitions of the central concept and associated concepts of the psycho-educational model

For the significance of the psycho-educational model, the central concept, to facilitate constructive management, is associated with the phenomenon of experienced aggression. To facilitate constructive management was defined as a process that the facilitator makes easier for the university lecturers. The facilitator assists the university lecturers to increase their effectiveness at managing experiences of aggression constructively. University lecturers’ increased effectiveness is helpful to them and promotes their development. With their promoted development and increased effectiveness, university lecturers should be able to deal with and control their experiences of aggression.

The following associated concepts were identified and defined in the context of the psycho-educational model.

#### Psycho-education

‘Psycho-education’ refers to education for persons who are living with emotive conflicts and difficulties and interventions involving a variety of activities that can educate and empower individuals to empower themselves. A psycho-educational approach proposes that, with self-knowledge of their own strengths, environmental resources and coping skills, individuals and groups are better equipped to deal with their difficulties and contribute to their own well-being (Lukens 2015:2–15; Reyes 2010:1). In this study, ‘psycho-education’ refers to the educational and developmental facilitation process of the psycho-educational model that assists and guides university lecturers to constructively manage experienced aggression.

#### Model

The most simplistic connotation of a model implies it to be a conceptual image or depiction that is implemented to help understand a phenomenon. A model is a symbolic illustration or an arrangement of knowledge of theoretical patterns in words, pictures and graphs and generally has three main measures: the input of information, the processing of information and the expected output of results (Chinn & Kramer 2015:251; SERC 2015:n.p.). In this study, a model was developed as a conceptual framework of reference to assist university lecturers to constructively manage their experiences of aggression through a facilitative process.

#### Experienced aggression

Aggression is any form of undesirable, negative behaviour that purposely harms another person. The behaviour is conducted with a specific intention and impacts the receiver’s happiness or engagements (Hershcovis, Reich & Niven 2015:9–11; Matthiesen & Einarsen 2010:202–248). In addition, aggression is any form of destructive behaviour with a specific negative aim in mind that can affect a person’s performance and accomplishments (Baron & Richardson 1994:7; Berkowitz 1993:20; Green 1990:4). In this study, aggression, concealed in frustration, is socially unacceptable and undesirable behaviour that negatively impacts people, causing potential emotional or professional harm to the person.

### Classification of concepts

The classification of concepts was based on the survey list of Dickoff et al. (1968). The classification included an agent, recipient, context, procedure, dynamics and terminus.

The agent is the person responsible for the initiation and facilitation of the process of commitment and interaction. In this psycho-educational model, the facilitator, as psycho-educator, is the agent who will facilitate the process and assist and enable university lecturers to deal with and control experienced aggression.

The recipient is the person(s) who will benefit from the engagement with the facilitator, as psycho-educator, and the facilitation process. The recipient in this psycho-educational model is the university lecturer from a diverse and multifaceted university who experiences aggression in his or her place of work.

The context is the environment or circumstance in which the facilitation for constructive management of experienced aggression occurs. The context for this psycho-educational model is a university, specifically a diverse and multifaceted university in South Africa. The university is the university lecturers’ workplace and reality, where the difficulty of experienced aggression occurs. The facilitative interaction is in the university context and in a group context.

The dynamics of this psycho-educational model are underscored by university lecturers’ experiences of aggression in their place of work. University lecturers experience a variety of forms of aggression in the workplace on levels of interaction with colleagues and students that have a negative personal and professional impact on them. University lecturers experience frustration and use unconstructive coping methods to navigate the phenomenon. The facilitator, as psycho-educator, is motivated to assist and support university lecturers in managing their experienced aggression constructively through a facilitation process to provide them with skills and develop them positively.

The process of this psycho-educational model, in association to the central concept, to facilitate constructive management, of experienced aggression by university lecturers, is a process whereby the psycho-educational facilitator makes it easier for university lecturers who experience aggression to address the problem. The psycho-educational facilitator also assists university lecturers to increase their effectiveness, which will be helpful to them in promoting their development. With their promoted development and increased effectiveness, university lecturers should deal with and control their experienced aggression. The facilitation will be implemented in three phases: the relationship phase, working phase and termination phase.

The terminus, or outcome, is the result of the facilitation process, and in this research the terminus is the constructive management of experienced aggression by enabled university lecturers.

### Relationship statements

- The relationship statements identified for this conceptual framework contribute to the understanding of the concepts in their entirety, instead of isolated individual descriptions (Chinn & Cramer 2015; Walker & Avant 2011). The suggested relationship statements of the identified concepts follow: facilitating the constructive management of experienced aggression is a process that the psycho-educational facilitator makes easier for university lecturers.
- The psycho-educational facilitator assists university lecturers to increase their effectiveness in managing experiences of aggression.
- University lecturers’ increased effectiveness is helpful to them and promotes their development.
- With their skills developed and increased effectiveness, university lecturers should deal with and control their experiences of aggression.

### Structure and process of the psycho-educational model

**FIGURE 1:**
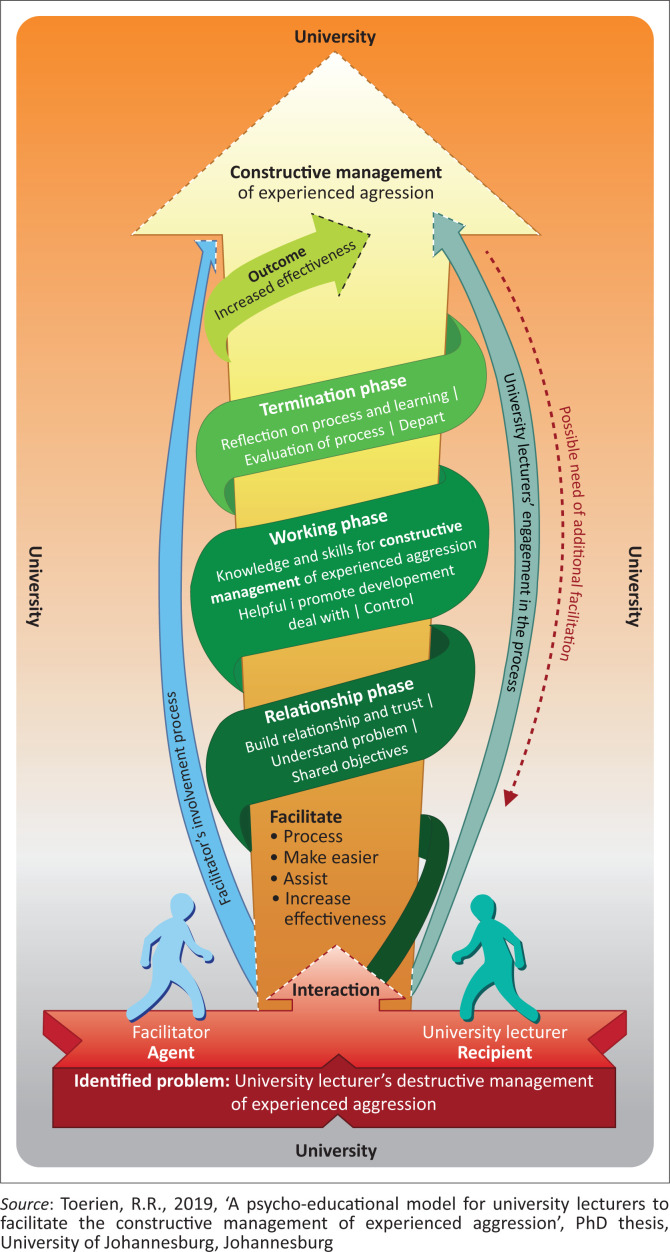
A psycho-educational model for university lecturers to facilitate the constructive management of experienced aggression.

A visual model was designed to describe the model’s structure and process and to illustrate the relationship between the concepts (see Figure 1). A description of the structure and process of the psycho-educational model follows.

#### Structure

The conceptual structure of the psycho-educational model was developed by implementing Chinn and Kramer’s (2015:186–208) criteria for the description and critical reflection of empirical theory. The main segments of the psycho-educational model included a rounded rectangle to represent the context of the psycho-educational model. The rounded rectangle has shades of grey that change into shades of orange, indicative of the aggression experiences and management of experiences, from destructive to constructive. The red, three-dimensional up-arrow call-out represents the problem, namely, destructive management of experienced aggression, and forms the basis of the psycho-educational model. The blue figurine represents the facilitator as psycho-educator (agent), and the wide blue arrow that curves upward to a narrow exit point represents the facilitator’s involvement in the facilitation process. The turquoise figurine represents the university lecturer (recipient), and the narrow turquoise arrow that curves upward to a wide exit point represents the university lecturer’s engagement in the facilitation process. The downward, dotted red arrow exiting from the recipient arrow represents the possibility of the additional need for facilitation by the recipient. The yellow upward arrow that gradually widens to the top represents the facilitation process and reflects the university lecturer’s development and growth throughout the process. The lightest green spiral segment represents the exit point of the facilitation phases. The arrow ends with a dotted arrow pointing to the final outcome of the facilitation process, which is represented by the colour white at the top of the yellow arrow. The final outcome of the psycho-educational model is the constructive management of experienced aggression by the participants. The upward green spiral represents the three phases of the facilitation process: (1) the relationship phase, (2) the working phase and (3) the termination phase as an integral and integrated part of the process that will be discussed below.

#### Process

The process of model development refers to the route, phases or overall patterns to the accomplishment of the goal (Dickoff et al. 1968). In this study, the psycho-educational model is implemented in three phases: the relationship phase, the working phase and the termination phase. These three phases stem from the shared interaction and participation between the facilitator and the university lecturers and form an integral part of the facilitation process and outcome of the process. These phases allow for individual development within a collaborative group dynamic, as well as divergence for additional facilitative assistance during the process because of university lecturers’ uniqueness and individuality.

**Phase 1: The relationship phase:** The main objective is to build trust and assist the university lecturers, to make it easier for them to participate in the facilitation process. The facilitator, university lecturers and individuals in the group must build a relationship of trust to strengthen confidence and information sharing. Trust is important for a group relationship and commitment to the group interaction in view of the facilitation process (Johnson 2009:41). To achieve this, it is vital to have a safe facilitative space and a psycho-educational climate that is conducive to discovery and self-directed learning in a learning climate that is open, dynamic and interactive. All individuals must come to a shared understanding of the phenomenon and a shared objective for the process. The facilitator must also stay cognisant that all university lecturers are unique individuals with their own realities, values and beliefs, when entering the relationship phase and throughout the entire facilitation process.

**Phase 2: The working phase:** The main objective of this phase is to facilitate the constructive management of experienced aggression and assist university lecturers to increase their effectiveness to do so. As a result, the working phase is characterised by the discovery of knowledge and skills that are helpful to university lecturers and that will promote their development on a personal and professional level to deal with and control their experiences of aggression. Four important skills were identified for the constructive management of experienced aggression: (1) intrapersonal competencies, (2) interpersonal competencies, (3) effective communication skills and (4) aggression (conflict) management skills (Toerien 2014). To effectively handle experienced aggression, university lecturers must understand the aggression phenomenon, engage in personal reflection and understanding of the self and their own behaviour, as well as acknowledge the negative impact of this behaviour (Vajda 2011:1–3). University lecturers must commit to exploring their negative handling of aggression and find solutions to the problem that would be helpful to them and that could promote their development in dealing with and controlling their experiences of aggression (Corey, Corey & Corey 2010:228). Increased effectiveness of the university lecturers’ intrapersonal, interpersonal and communication knowledge and skills will assist them in effectively dealing with and controlling experienced aggression and conflict in their places of work. Gallo (2014:n.p.) posits that a person who experiences aggression should take action.

**Phase 3: The termination phase:** The main objective is to reflect on the process and personal growth of the university lecturers during the implementation of the model in a facilitative workshop. The participants must reflect on whether their shared objectives were met. This reflection increases the participants’ skill and effectiveness at implementing the model in practice in their workplace. The facilitator’s involvement is minimal in this phase: to reinforce the knowledge and skills that the participants attained during the facilitation process. The participants also engage in self-reflection and individual journal writing to further reinforce the helpful knowledge and skills obtained, namely, increased effectiveness at managing experienced aggression constructively.

### Evaluation of the model

The psycho-educational model was evaluated by implementing the criteria for model evaluation described by Chinn and Kramer (2015:199–208). The criteria are clarity, simplicity, generality, accessibility and importance. The model was presented to a panel of academics who were experienced model developers and model evaluators from the Faculty of Education and the Faculty of Health Sciences at a university in South Africa. The panel comprised six experts, four with PhDs and two professors, who have supervised well over 100 PhD candidates. The panel further included two doctoral candidates in the final stages of model development. The panel members completed a written evaluation, followed by an in-depth and critical discussion of the developed psycho-educational model. The evaluation panel agreed that the psycho-educational model’s concept analysis and relationship statement were clearly defined and that the structure of the psycho-educational model was clear, meaningful and helpful. The interactiveness of the process was also found to be clear and simplistic. The criterion of simplicity, which focussed on the number of structural elements within each descriptive category of the model and the relationship within the theory, was found to be simple and the relationship statements to be understandable and easy to follow. The scope of the research was within a specific, identified context, namely, a diverse and multicultural South African university. The psycho-educational model was conferred as valuable and applicable to a wider education, workplace and human resources context than a university context. This aspect was reiterated after the presentation of the psycho-educational model at an international health conference hosted in South Africa in March 2018. The concepts of the model were described by conference attendees as adequately general, accessible and applicable to other groups of people and other human resources management processes. The psycho-educational model was described as important, significant and of value in individuals’ personal and professional development and growth. The evaluation pointed out that the psycho-educational model could bring about change and increased effectiveness in university lecturers’ ability to constructively manage experiences of aggression. In conclusion, university lecturers’ development and increased effectiveness may contribute to improved work environments, interactions and relationships. Next, the original contributions of the psycho-educational model will be described.

## Original contribution of the study

The research makes a creative and distinctive contribution to education and psycho-education. The study has theoretical as well as practical value in the field of psycho-education, and for universities and higher education overall. Hence, it is recommended that university lecturers’ experiences of aggression should be acknowledged as a reality, and therefore training and development of knowledge and skills by university lecturer’s to constructively manage experiences of aggression constructively is essential.

## Limitations of the study

It is important to recognise that there are limitations to the study, because the study only included a specific faculty at a university and not all faculties at a university. The implementation of the model in a workshop and in practice, and the evaluation of the model implementation, have not yet been published.

## Recommendations

The psycho-educational model is vested in the assumption that with facilitation, university lecturers’ effectiveness at managing experienced aggression constructively can be increased. Hence, it is recommended that university lecturers’ experiences of aggression should be acknowledged as a reality, and therefore training and development of knowledge and skills to manage experiences of aggression constructively is essential. The recommendations for education and psycho-education are that (1) the model could be applied in educating and training line managers overseeing junior faculty; (2) the model could be applied in educating and training human resource professionals to improve and increase their ability to effectively deal with and support university lecturers who experience aggression in their place of work; (3) the model could be introduced to all new university lecturers as part of their induction to a university career to promote their development and increase their effectiveness; and (4) the model could be applied in educating and training facilitators as psycho-educators to understand the phenomena of aggression and destructive management of experienced aggression.

## Conclusion

The purpose of this research was to develop, describe and evaluate a psycho-educational model as a conceptual framework for university lecturers to facilitate the constructive management of experienced aggression. The researchers believe that the research purpose was achieved by in-depth description of the concepts, their relationship statements and the description of the steps of model development, as well as adherence to ethical principles and trustworthiness. The implementation of the model in a workshop and in practice, and the evaluation of the implementation, will be discussed in a follow-up article.
